# Levodopa/carbidopa/entacapone versus levodopa/benserazide plus pramipexole in Chinese patients with Parkinson’s disease experiencing wearing off

**DOI:** 10.3389/fneur.2025.1682614

**Published:** 2025-11-19

**Authors:** Cuiyu Yu, Weiguo Liu, Aiping Gong, Min Ye, Hui Huang, Yang Zhao, Chunfeng Liu, Yijing Guo, Juping Chen, Xueling Zhang, Xianwen Chen, Lihua Shen, Dan Li

**Affiliations:** 1 Department of Neurology, Nanjing Brain Hospital Affiliated to Nanjing Medical University, Nanjing, Jiangsu, China; 2 Department of Neurology, The Second Affiliated Hospital of Xuzhou Medical University, Xuzhou, Jiangsu, China; 3 Department of Neurology, BENQ Medical Center, Nanjing, Jiangsu, China; 4 Department of Neurology, Huaibei People’s Hospital, Huaibei, Anhui, China; 5 Department of Neurology, Nanjing Hospital of Chinese Medicine Affiliated to Nanjing University of Chinese Medicine, Nanjing, Jiangsu, China; 6 Department of Neurology, The Second Affiliated Hospital of Soochow University, Soochow, Jiangsu, China; 7 Department of Neurology, Affiliated Zhongda Hospital of Southeast University, Nanjing, Jiangsu, China; 8 Department of Neurology, Changshu Hospital Affiliated to Nanjing University of Chinese Medicine, Changshu, Jiangsu, China; 9 Department of Neurology, The Affiliated Suqian Hospital of Xuzhou Medical University, Suqian, Jiangsu, China; 10 Department of Neurology, The First Affiliated Hospital of Anhui Medical University, Hefei, Anhui, China; 11 Department of Neurology, Affiliated Hospital of Nantong University, Nantong, Jiangsu, China; 12 Department of Neurology, Jiangsu Province (Suqian) Hospital, Suqian, Jiangsu, China

**Keywords:** Parkinson’s disease, wearing off, levodopa/carbidopa/entacapone, pramipexole, Chinese patients

## Abstract

**Objective:**

To compare the efficacy and safety of direct switch from levodopa/benserazide (LB) to levodopa/carbidopa/entacapone (LCE) versus LB plus pramipexole (PPX) in Chinese patients with Parkinson’s disease (PD) experiencing wearing off (WO).

**Methods:**

In this multicenter, prospective, randomized, open-label, observational study, 140 patients with PD experiencing WO who had been on stable LB treatment were enrolled and randomized 3:2 to receive LCE (84) or LB + PPX (56) treatment for 8 weeks. The primary endpoint was change in the daily “OFF” time from baseline. Change in the daily “ON” time was also assessed. Treatment-emergent adverse events (TEAEs) were recorded.

**Results:**

Seventy-nine patients in the LCE group and 49 patients in the PPX group completed the study. Both LCE and PPX shortened the patients’ daily OFF time significantly after 8 weeks (−1.76 ± 1.70 h, *p* < 0.001 and −1.51 ± 1.60, *p* < 0.001, respectively), and the shortenings were comparable between the two groups (*p* = 0.414). Correspondingly, both the LCE group and the PPX group had significantly increased daily ON time (1.62 ± 1.59 h, *p* < 0.001 and 1.38 ± 1.65, *p* < 0.001, respectively), and the increases were comparable between the two groups (*p* = 0.412). Both treatments improved the patients’ WO symptoms, sleep quality, depression and quality of life. Six (7.59%) patients in the LCE group and 7 (14.29%) patients in the PPX group reported TEAEs, all of which were mild and tolerable. One patient in the LCE group and 2 patients in the PPX group experienced mild dyskinesia.

**Conclusion:**

LCE and LB + PPX were both effective, safe and tolerable in treating patients with PD who experienced WO.

## 1 Introduction

Parkinson’s disease (PD), a progressive neurodegenerative disorder, has a prevalence of about 1.7% in people aged 65 years or older in China (1–6). In addition, it is estimated that in 2030, China will have close to half of the PD population in the world (5, 6). Levodopa remains the “gold standard” treatment for motor symptoms of PD (1, 2). Conventional levodopa formulations incorporate a dopa-decarboxylase (DDC) inhibitor (DDCI) such as carbidopa and benserazide to prevent peripheral conversion of levodopa into dopamine (7, 8). Despite excellent response to levodopa/DDCI in the early stage, patients receiving long-term levodopa treatment often develop motor complications such as wearing off (WO) and dyskinesia (4, 7). Patients with WO experience re-emergence or worsening of Parkinsonian symptoms before the next scheduled dose of levodopa (“OFF period”) and WO may be accompanied by peak-dose dyskinesia (1, 4, 7). These OFF periods can get worse over time, leading to impaired mobility and decreased quality of life (QoL) (1). About 45% of the patients experience WO within 5 years after initiating levodopa treatment and almost all of the patients taking 10 years of levodopa have motor complications (4, 8). WO is mainly caused by the loss of dopamine-producing neurons in the substantia nigra (SN) that leads to diminished buffering potential against the fluctuation of plasma levodopa, as levodopa has short half-life (1, 9, 10). As a result, SN delivers dopamine to the striatum in an intermittent, pulsatile pattern rather than the normal tonic and continuous manner, and deep troughs in plasma levodopa is increasingly translated into corresponding deep troughs in striatal levodopa (4, 9, 10).

Levodopa/carbidopa/entacapone (LCE) is an optimized levodopa formulation that inhibits both DDC and catechol-O-methyltransferase (COMT), two enzymes important in levodopa metabolism (1, 4). Therefore, LCE inhibits peripheral levodopa metabolism and increases the amount of levodopa reaching the brain (1, 4). Carbidopa increases the plasma half-life of levodopa from 50 min to 1.5 h, and entacapone, a peripheral-acting COMT inhibitor, further increases its half-life to 2.4 h (1, 4). In addition, entacapone decreases its peak-trough variation by 30% and increases its bioavailability by approximately 35% (1, 4). Numerous studies found that in levodopa/DDCI-treated patients with WO, entacapone increased their daily ON time by 1–2 h, reduced their daily OFF time correspondingly and improved their UPDRS scores at a reduced levodopa daily dose (1, 4, 11–15). In addition, the benefits were maintained over several years (15). Studies have confirmed that LCE provides clinical benefits equivalent to levodopa/DDCI plus entacapone and that direct switch from levodopa/DDCI to LCE was effective and safe in treating patients with WO (1, 16). In China, LCE is increasingly prescribed for patients with WO. However, there has been no published study of its efficacy and safety in treating Chinese patients. In the current study, we compared the efficacy, safety and tolerability of direct switch from levodopa/benserazide (LB) to LCE in Chinese patients experiencing WO with LB plus pramipexole (PPX), a non-ergoline dopamine receptor agonist (DA) commonly used to treat patients with WO (17). Chinese patients often take PPX at a lower dose than those recommended by the Chinese PD consensus as well as than patients in the western countries (17, 18). Whether low-dose PPX was effective in treating patients with WO is also a question of interest. Our study is the first study that compared LCE with PPX in treating patients with WO and such a study can help neurologists in China in their effort to choose a proper treatment for patients with WO.

## 2 Materials and methods

### 2.1 Study design and patients

This multicenter, prospective, randomized, open-label, observational study was carried out in 12 hospitals in China (Supplementary Text 1). The study was conducted in accordance with the principle of the Declaration of Helsinki and was approved by the Institutional Review Board of Nanjing Brain Hospital (approval number: 2020-KY140-01). Written informed consent to participate in the study was obtained from all participants before screening.

The study consisted of a 1-week screening period and an 8-week treatment period, wherein the treatment period consisted of a 4-week titration period followed by a 4-week maintenance period.

Inclusion criteria: (1) Male or female patients aged 30–80 years diagnosed with idiopathic PD according to the 2015 Movement Disorder Society (MDS) clinical diagnostic criteria for Parkinson’s disease (19) who experienced WO, wherein WO was defined as complaint of dose-related motor fluctuations and at least one positive symptom in the Wearing-Off Questionnaire (WOQ)-19 (20), (2) on stable LB treatment (no dose change within 4 weeks before enrollment) at a levodopa equivalent daily dose (LEDD) ≥ 300 mg, (3) ≥ 1.5 h daily “OFF” time; (4) a Hoehn and Yahr (H&Y) stage of 1.5–4, and (5) had not received entacapone or DA treatment within 1 months before enrollment.

Exclusion criteria: (1) Had atypical Parkinsonism’s (Parkinsonism-plus syndrome), (2) had surgery within 6 months before the study, (3) had uncontrolled severe hypertension (systolic blood pressure ≥180 mmHg), (4) had severe cerebral arteriosclerosis or cerebrovascular disease-associated limb dysfunction, (5) alcoholics, drug addicts, or patients with severe cognitive impairment (including severe Alzheimer’s disease) who were unable to comply with the treatment and examination (Mini-Mental State Examination [MMSE] < 24), (6) Beck Depression Inventory (BDI) < 17, (7) had mental disorders, epilepsy, being pregnant or lactating, (8) had severely impaired cardiac, liver or renal function, joint diseases or other condition(s) that would affect efficacy assessment in the study, (9) had participated in other clinical studies within 2 months before the current study, (10) had taken entacapone or DA within 4 weeks before enrollment, (11) had dyskinesia, (12) abnormal laboratory results: white blood cells (WBC) < 3.0 × 10^9^/L, platelets <80 × 10^9^/L, hemoglobin < 80 g/L, alanine aminotransferase (ALT) > 2.5 times the normal range, or creatinine >1.5 times the normal range, or (13) abnormal electrocardiogram (ECG) reading such as clinically meaningful prolonged QT intervals, ventricular tachycardia, atrial fibrillation and heart block.

### 2.2 Randomization and treatment

All of the enrolled patients were randomized 3:2 using central randomization to receive LCE (the “LCE” group) or LB plus PPX (the “PPX” group), respectively.

The study medication in the LCE group was LCE 100 mg/25 mg/200 mg (Stalevo 100, Eisai Co., Ltd., Tokyo, Japan). As a general principle, levodopa dose and dosing frequency remained unchanged after the switch. Specifically, patients who had been taking 1/2 tablet of LB 200 mg/50 mg (Madopar 250, Roche, Basel, Switzerland) per dose switched directly to one tablet of Stalevo 100 per dose, and patients who had been taking one tablet of Madopar 250 per dose switched to one tablet of Stalevo 100 plus 1/2 tablet of Madopar 250 per dose. For those patients who had been taking LB > 3 times a day, switches to LCE for all doses should be completed by Week 3. Schedule of switching to LCE for patients whose daily levodopa dose was >300 mg (but ≤600 mg) was described in Supplementary Table 1. In addition, during the titration period, increasing LCE dosing frequency (3–5 times a day) was the first choice for those patients who needed levodopa dose increase, while LB dose reduction was the first choice for those patients who needed levodopa dose reduction. Finally, Levodopa daily dose was between 100 mg-750 mg.

The study medication in the PPX group was pramipexole (immediate-release tablets 0.25 mg). Patients in the PPX group received PPX at an initial dose of 0.125 mg three time a day (tid) in addition to their baseline LB. For those patients who experienced adverse reactions, the dosing frequency of PPX was reduced to 1–2 times a day. The dose of PPX was up-titrated at increments of 0.125 mg at one-week intervals based on the patients’ response during the titration period and the acceptable range of maintenance PPX daily dose was 0.125 mg-1.5 mg. PPX dose reduction during Weeks 3–4 was allowed.

Use of benzoxol, amantadine and/or monoamine oxidase B (MAO-B) inhibitors was allowed, and their daily dose remained the same during the study.

All participating patients purchased the medications prescribed by their neurologists, all of which are covered by the national medical insurance.

### 2.3 Data collection

Demographic information and family history were collected from all of the patients during the screening visit. During the screening visit and the follow-up visit after 8 weeks of treatment, vital signs were recorded, and ECG and laboratory tests were performed for all participants. Also, during the screening visit and the follow-up visit, every patient completed the following questionnaires/scales: (1) WOQ-19, (2) MDS-Unified Parkinson’s Disease Rating Scale (MDS-UPDRS), (3) The Parkinson’s Disease Sleep Scale-2 (PDSS-2), (4) MMSE, (5) Modified versions of the Abnormal Involuntary Movement Scale (mAIMS), (6) Epworth Sleepiness Scale (ESS), and (7) the Parkinson’s Disease Questionnaire (PDQ-39).

Every participant completed a standardized home diary for the 3 days before their switch to LCE or to LB + PPX as well as for the 3 days before their follow-up visit after 8 weeks of treatment. During the 3 days, the patients recorded in the diary whether they were “ON,” “OFF,” “ASLEEP,” “ON with mild dyskinesia” or “ON with severe dyskinesia” at half-hour intervals. If a patient experienced more than one state within a half-hour interval, the state that lasted the longest was recorded. The patients were trained to fill in the home diary properly before they started the baseline home diary and were asked to set up reminders on their cellphone.

### 2.4 Efficacy endpoints

The primary endpoint was change in the daily “OFF” time from baseline after 8 weeks of treatment based on information collected from the home diaries. Secondary endpoints included: (1) change in the daily “ON” time from baseline, (2) response to the treatment in the WO symptoms according to the WOQ-19, (3) change in the MDS-UPDRS, Part 1 non-motor aspects of experiences of daily living [nM-EDL] from baseline, (4) change in the PDQ-39 score (14, 21), (5) change in the PDSS-2 score (22), (6) change in the BDI score (23), and (7) the 7-point Clinical Global Impression of Change (CGI-C) (16, 21) after 8 weeks of treatment assessed by the patients.

### 2.5 Safety and tolerability

Patients’ vital signs, ECG, laboratory test results were recorded. Treatment-emergent adverse events (TEAEs) and their severity were recorded.

### 2.6 Statistical analyses

The intended sample size was based on a minimal clinically important change (mean changes in actively treated subjects rated minimally improved on CGI-I) of 1.0 h in the daily “OFF” time and a non-inferiority margin of 1.2 h in the daily “OFF time” (12, 24, 25). Three hundred and fifty-four patients (212 in the LCE group and 142 in the PPX group) were needed to have a statistical power of 80% for a one-sided test with a significance level of 0.025. Assuming a dropout rate of 10%, a total of 390 patients (234 in the LCE group and 156 in the PPX group) were planned. However, as the recruitment process was extremely slow and finally halted during the height of the COVID pandemic, 140 patents (84 in the LCE group and 56 in the PPX group) were actually enrolled.

SPSS 18.0 (IBM, Armonk, NY, United States) was used to perform all statistical analyses in the study. Efficacy analyses were performed in the full analysis set (FAS) (all patients who received at least one dose of study medication and had at least one post-dosing efficacy assessment). Safety analyses were performed in the safety set (SS) (all patients who received at least one dose of study medication and had at least one post-dosing safety assessment). For the 3-day home diary-derived values, average of data from the 3 days were calculated. If one of the 3 days contained missing data, average of data from the remaining 2 days were calculated. If two of the 3 days had missing data, data from the remaining 1 day were used. Patients whose follow-up diary had missing data for all of the 3 days were considered to be lost to follow-up and not included in the FAS. Patients whose baseline diary had missing data for all of the 3 days were not enrolled. Descriptive statistics was used. Categorical variables were expressed as *N* (%) and continuous variables were expressed as means ± standard deviations (SD) or means (minimum, maximum). The student *t* test and the paired *t* test were used for intergroup and intragroup comparisons of the daily “OFF” time, “ON” time and LEDD, respectively. Non-parametric independent sample *t*-test was used for intergroup comparisons of changes in the 13 sub-scores of the MDS-UPDRS Part 1. Analysis of covariance (ANCOVA) and the paired *t* test were used for intergroup and intragroup comparisons of the PDQ-39, PDSS-2 and BDI scores, respectively. The Wilcoxon rank-sum test was used for intergroup comparison of CGI-C. Statistical significance was achieved with a *p*-value of <0.05.

## 3 Results

### 3.1 Demographics and baseline clinical characteristics

**Figure 1:**
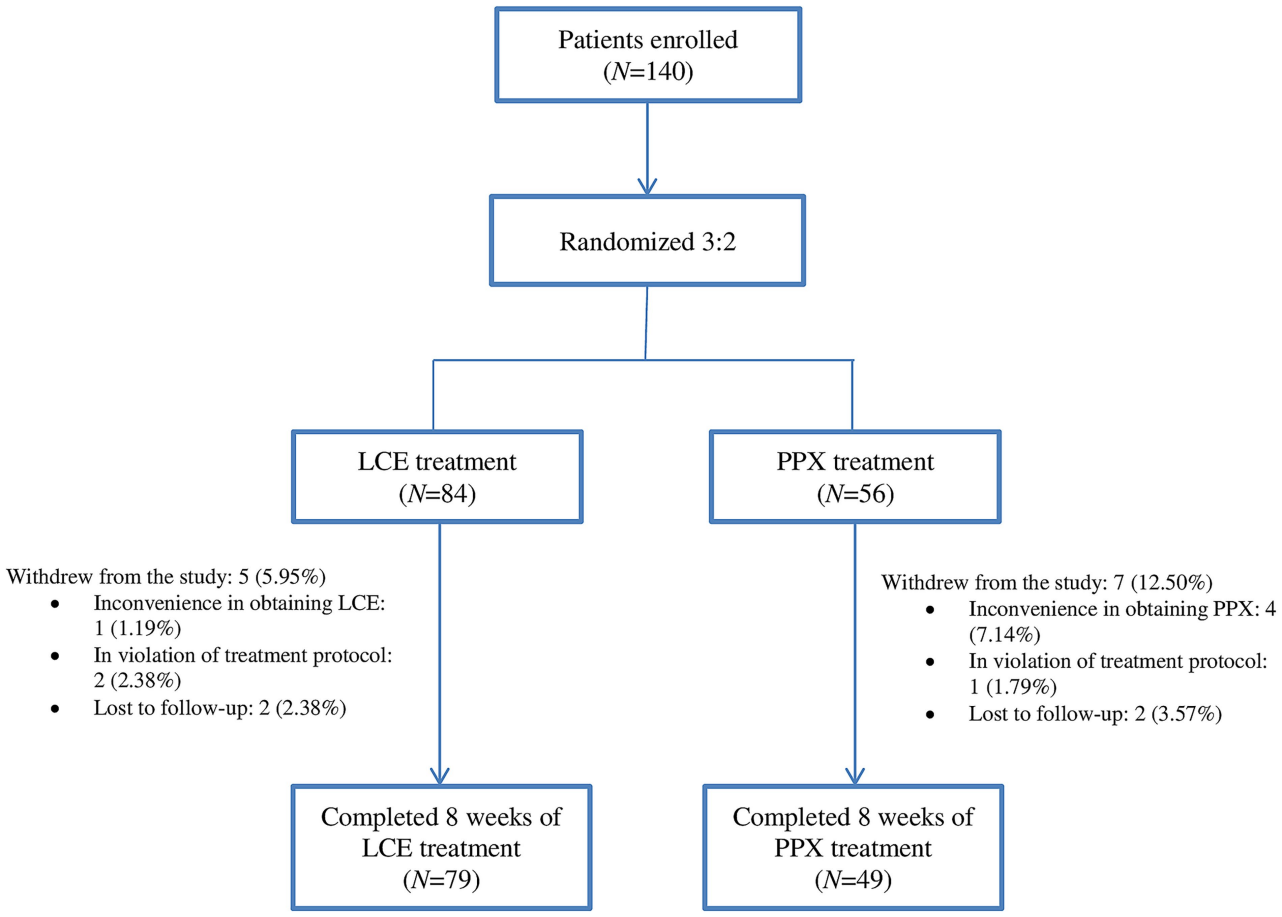
Study flow chart: the number of enrolled patients (*N*), their randomization (*N*), the number of patients who completed the study (*N*), and reasons for withdrawal from the study. LCE, levodopa/carbidopa/entacapone; PPX, pramipexole.

Study flow chart was illustrated in Figure 1. A total of 140 patients were enrolled and randomized 3:2 to received LCE (84) and LC + PPX (56), respectively. Five (5.95%) patients in the LCE group and 7 (12.50%) patients in the PPX group withdrew from the study without taking a dose of study medication or having a post-dosing efficacy/safety assessment. Seventy-nine (94.05%) patients in the LCE group and 49 (87.50%) patients in the PPX group completed the study and they constituted FAS and SS. Patient demographics and baseline characteristics were described in Table 1. The 128 patients who completed the study had a mean age of 67.17 ± 8.83 years and 68 (53.13%) of them were male. Their mean duration of PD was 8.35 ± 4.20 years. The two groups of patients had comparable demographics, age at PD onset and duration of PD. In addition, the LCE group and the PPX group had comparable baseline LEDD (445.85 ± 164.05 mg vs. 486.22 ± 205.78 mg, *p* = 0.222), daily OFF time (5.47 ± 2.65 h vs. 5.95 ± 2.61 h, *p* = 0.309), daily ON time (9.30 ± 1.92 h vs. 9.45 ± 2.42 h, *p* = 0.704), BDI scores (6.34 ± 7.14 vs. 8.18 ± 4.32, *p* = 0.071) and PDQ-39 scores (28.23 ± 27.51 vs33.84 ± 19.20, *p* = 0.179). However, the LCE group had a significantly lower baseline PDSS-2 score than the PPX group (13.09 ± 13.24 vs. 18.43 ± 10.50, *p* = 0.018) (Table 1).

**Table 1:** Patient demographics and baseline clinical characteristics (FAS).

Characteristics	All patients (*N* = 128)	LCE (*N* = 79)	PPX (*N* = 49)	*p*-value
Gender				0.230
Male	68 (53.13%)	44 (55.70%)	24 (48.98%)	
Female	60 (46.88%)	35 (44.30%)	25 (51.02)	
Age, years	67.17 ± 8.83	68.1 ± 8.55	65.67 ± 9.14	0.131
Age at PD onset, years	58.75 ± 9.57	59.95 ± 8.93	56.81 ± 10.33	0.077
Duration of PD, years	8.35 ± 4.20	8.03 ± 3.76	8.87 ± 4.83	0.309
LEDD, mg	461.30 ± 181.45	445.85 ± 164.05	486.22 ± 205.78	0.222
“On” time, hours	9.36 ± 2.12	9.30 ± 1.92	9.45 ± 2.42	0.704
“Off” time, hours	5.65 ± 2.64	5.47 ± 2.65	5.95 ± 2.61	0.309
PDSS-2	15.13 ± 12.50	13.09 ± 13.24	18.43 ± 10.50	**0.018**
BDI	7.05 ± 6.26	6.34 ± 7.14	8.18 ± 4.32	0.071
PDQ-39	30.39 ± 24.71	28.23 ± 27.51	33.84 ± 19.20	0.179

Categorical variables were expressed as *N* (%) and continuous variables were expressed as mean ± standard deviation. Bold values indicate statistical significance at the level of *p* < 0.05. FAS, full analysis set; LCE, Levodopa/Carbidopa/Entacapone; PPX, Pramipexole; PD, Parkinson’s disease; LEDD, levodopa-equivalent daily dose; PDSS-2, Parkinson’s disease sleep scale; BDI, Beck’s Depression Inventory; PDQ-39, Parkinson’s Disease Questionnaire-39.

**Figure 2:**
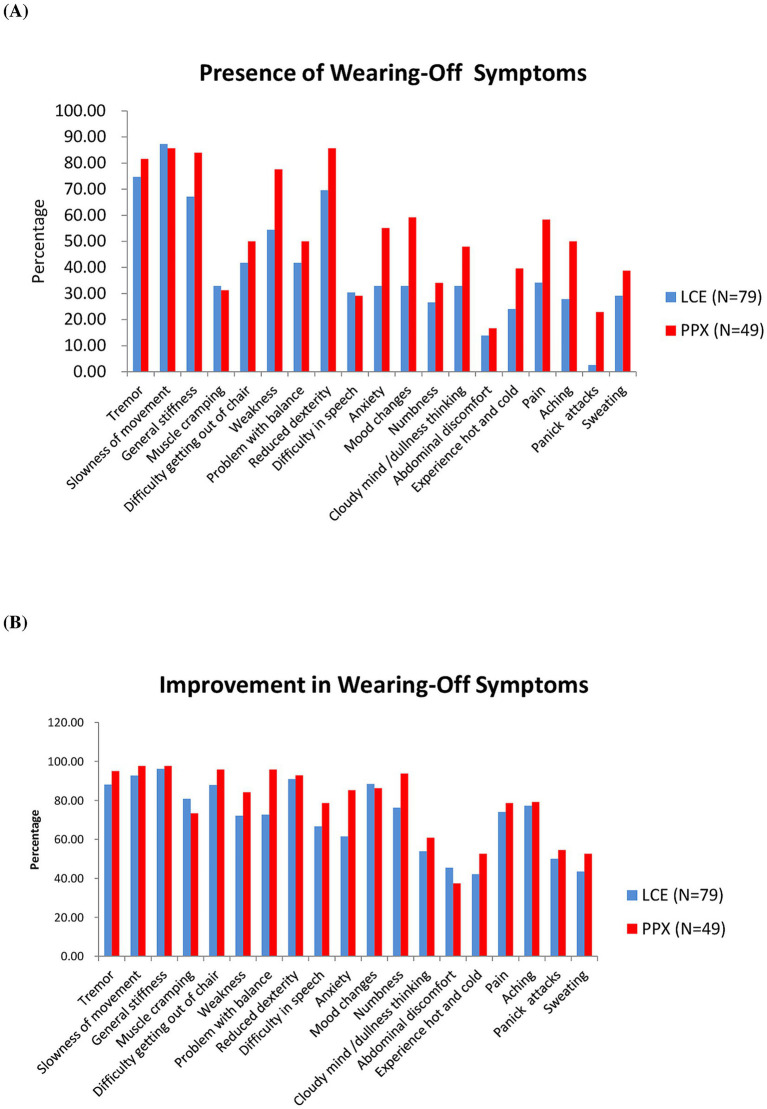
Wearing-Off Questionnaire-19. **(A)** Presence of wearing-off symptoms in the two groups of patients at baseline (%). **(B)** Improvement in wearing-off symptoms in the two groups of patients after 8 weeks of treatment (%). LCE, levodopa/carbidopa/entacapone; PPX, pramipexole.

According to the WOQ-19, the most common motor WO symptoms for patients in the LCE and in the PPX groups were slowness of movement (87.34 and 85.71%,), tremor (74.68 and 81.63%), general stiffness (67.09 and 84.00%) and reduced dexterity (69.62 and 85.71%), and the least common ones were difficulty in speech (30.38 and 29.17%) and muscle cramping (32.91 and 31.25%) (Figure 2A). Meanwhile, the most common non-motor WO symptoms for the LCE group and the PPX group were anxiety (32.91 and 55.10%), mood change (32.91 and 59.18%), pain (34.18 and 58.33%) and numbness (26.58 and 34.04%), and the least common ones were abdominal discomfort (13.92 and 16.67%) and panic attack (2.53 and 22.92%) (Figure 2A).

### 3.2 Changes in the daily OFF time and ON time after 8 weeks of treatment

Both LCE treatment and LB + PPX treatment shortened the patients’ daily OFF time significantly after 8 weeks (−1.76 ± 1.70 h, *p* < 0.001 and −1.51 ± 1.60, *p* < 0.001, respectively), and the shortenings were comparable between the two treatments (*p* = 0.414) (Table 2).

**Table 2:** Changes in efficacy outcomes and LEDD after 8 weeks of treatment (FAS).

Outcomes	LCE (*N* = 79)				PPX (*N* = 49)				*p*-value^**^
	Baseline	Week 8	Change	*p*-value^*^	Baseline	Week 8	Change	*p*-value^*^	
“On” time, hours	9.30 ± 1.92	10.92 ± 2.20	1.62 ± 1.59	**<0.001**	9.45 ± 2.42	10.83 ± 2.32	1.38 ± 1.65	**<0.001**	0.412
“Off” time, hours	5.47 ± 2.65	3.70 ± 2.80	−1.76 ± 1.70	**<0.001**	5.95 ± 2.61	4.44 ± 2.44	−1.51 ± 1.60	**<0.001**	0.414
PDQ-39	28.23 ± 27.51	22.50 ± 23.57	−5.66 ± 12.95	**<0.001**	33.84 ± 19.20	28.35 ± 17.15	−5.49 ± 10.40	**0.001**	0.939
PDSS-2	13.09 ± 13.24	9.42 ± 9.13	−3.67 ± 8.90	**<0.001**	18.43 ± 10.50	13.84 ± 8.36	−4.59 ± 6.27	**<0.001**	0.528
BDI	6.34 ± 7.14	5.19 ± 6.37	−1.15 ± 2.67	**<0.001**	8.18 ± 4.32	6.10 ± 4.90	−2.08 ± 4.43	**0.002**	0.140
LEDD, mg	445.85 ± 164.05	620.03 ± 218.74	174.18 ± 161.97	**<0.001**	486.22 ± 205.78	580.08 ± 187.25	93.86 ± 60.94	**<0.001**	**<0.001**

Variables were expressed as means ± standard deviation. Bold values indicate statistical significance at the level of *p* < 0.05. ^*^ *p*-value for intragroup comparison of Week 8 with Baseline. ^**^ *p*-value for intergroup comparison of changes from baseline between LCE and PPX. LEDD, levodopa-equivalent daily dose; FAS, full analysis set; LCE, levodopa/carbidopa/entacapone; PPX. pramipexole; PD, Parkinson’s Disease.

Significantly increased daily ON time was also observed in both the LCE group and the PPX group after 8 weeks of treatment (1.62 ± 1.59 h, *p* < 0.001 and 1.38 ± 1.65, *p* < 0.001, respectively), and the increases were comparable between the two groups (*p* = 0.412) (Table 2).

### 3.3 Improvement in WO symptoms

According to the WOQ-19, LCE and LB + PPX were both very effective in improving the patients’ motor WO symptoms (Figure 2B). More than 80% of the patients in the LCE and in the PPX groups reported improvements in slow of movement (92.75 and 97.63%), general stiffness (96.23 and 97.62%), reduced dexterity (90.91 and 92.86%), tremor (88.14 and 95.00%) and difficulty getting out of chair (87.88 and 95.83%). Rates of improvement in difficulty in speech and muscle cramping were the lowest among the motor WO symptoms for the LCE group (66.67%) and the PPX group (73.33%), respectively (Figure 2B).

As to the non-motor WO symptoms, more than 60% of the patients in the LCE and the PPX groups reported improvements in mood change (88.46 and 86.21%), numbness (76.19 and 93.75%), pain (74.07 and 78.57%), aching (77.27 and 79.17%) and anxiety (61.54 and 85.19%), and the two treatments were less effective in improving the other 5 non-motor WO symptoms (Figure 2B).

### 3.4 Changes in LEDD, PDQ-39, PDSS-2 and BDI after 8 weeks of treatment

LEDD for both the LCE group and the PPX group were significantly increased (174.18 ± 161.97 mg, *p* < 0.001, and 93.86 ± 60.94 mg, *p < 0.001*, respectively), and the LCE group had significantly greater LEDD increase than the PPX group (*p* < 0.001) (Table 2).

Both the LCE group and the PPX group had significantly decreased PDQ-39, PDSS-2 and BDI scores after 8 weeks of treatment (*p* all <0.001), and the decreases in the PDQ-39, PDSS-2 and BDI scores were all comparable between the two treatment groups (*p* = 0.939, *p* = 0.528 and *p* = 0.140, respectively) (Table 2).

### 3.5 Change in the MDS-UPDRS, Part 1 non-motor aspects of experiences of daily living

Both treatments led to insignificant changes in all of the 13 sub-scores of the MDS-UPDRS, Part 1, and the changes in 12 of them were comparable between the 2 treatments. However, there was significant difference in changes in the hallucination and psychosis sub-score between the two treatments (Table 3). Specifically, the hallucination and psychosis sub-score decreased slightly in the LCE groups and increased slightly in the PPX group (Table 3).

**Table 3:** Changes in MDS-UPDRS Part I from baseline after 8 weeks of treatment (FAS).

Items	LCE (*N* = 79)	PPX (*N* = 49)	*p*-value
Cognitive impairment	−0.14 (−2, 1)	−0.19 (−2, 1)	0.624
Hallucination and psychosis	−0.09 (−2, 0)	0.11 (0, 1)	**0.001**
Depressed mood	−0.13 (−1, 1)	−0.17 (−2, 1)	0.846
Anxious mood	−0.10 (−2, 2)	−0.18 (−2, 1)	0.413
Apathy	−0.09 (−2, 1)	−0.16 (−1, 2)	0.162
Features of dopamine dysregulation syndrome	−0.05 (−3, 2)	−0.05 (−3, 3)	0.863
Sleep problems	−0.24 (−2, 3)	−0.40 (−2, 2)	0.262
Daytime sleepiness	−0.08 (−2, 1)	−0.13 (−2, 2)	0.489
Pain and other sensations	−0.17 (−3, 2)	−0.39 (−2, 1)	0.079
Urinary problems	−0.14 (−2, 1)	−0.04 (−2, 1)	0.228
Constipation problems	−0.09 (−2, 3)	−0.10 (−2, 2)	0.831
Light headedness on standing	−0.01 (−1, 1)	0.02 (−2, 1)	0.536
Fatigue	−0.15 (−2, 1)	−0.22 (−2, 1)	0.477

Values were expressed as means (minimum, maximum). Bold values indicate statistical significance at the level of *p* < 0.05. MDS-UPDRS, MDS-Unified Parkinson’s Disease Rating Scale; FAS, full analysis set; LCE, levodopa/carbidopa/entacapone; PPX, pramipexole.

### 3.6 Clinical global impression of change

**Figure 3:**
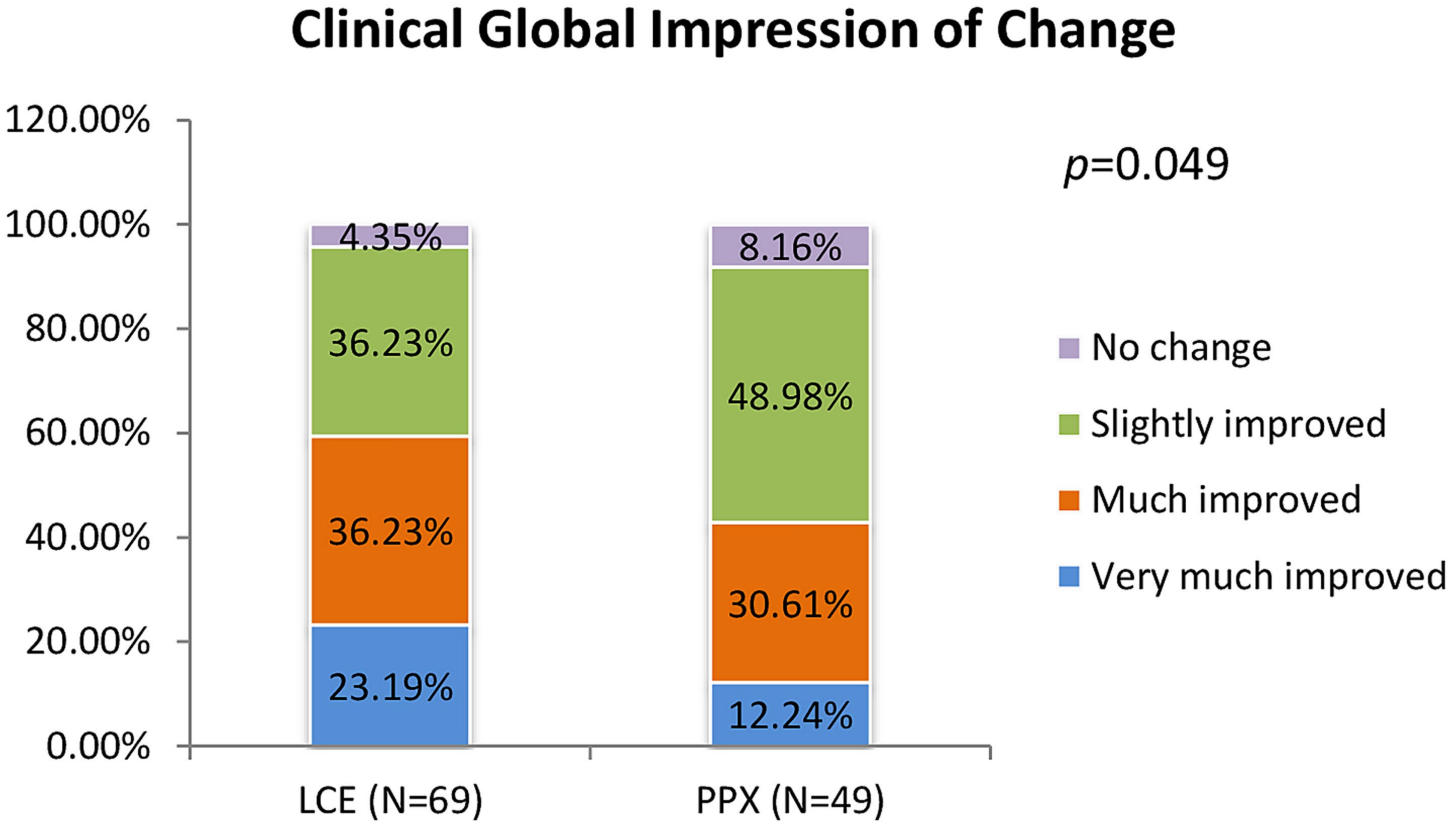
Clinical global impression of change after 8 weeks of treatment (% of the patients who very much improved, much improved, slightly improved and had no change). LCE, levodopa/carbidopa/entacapone; PPX, pramipexole.

After 8 weeks of treatment, 95.65% of the patients in the LCE group and 91.84% of the patients in the PPX group reported improvement. There was significant difference in distributions of patients reporting different degrees of improvement between the two groups (*p* = 0.049). The percentages of patients reporting “very much improved” and “much improved” were higher in the LCE group (23.19 and 36.23%) than in the PPX group (12.24 and 30.61%), and the percentages of patients reporting “slightly improved” and “no change” were lower in the LCE group (36.23 and 4.35%) than in the PPX group (49.98 and 8.16%) (Figure 3). No patients reported worsening of their symptoms.

### 3.7 Safety and tolerability

Six (7.59%) patients in the LCE group and 7 (14.29%) in the PPX reported at least one TEAEs. There was no significant difference in the incidence of TEAEs between the two groups (*p* = 0.223). The most common TEAE in the LCE group was urine discoloration (3 [3.80%]), In addition, one patients experienced mild dyskinesia that resolved after reducing LCE dosing frequency to 1–2 times a day. Another patient experienced insomnia that also resolved after taking LCE only in the morning and noon.

The most common TEAEs in the PPX group were dyskinesia (2 [4.08%]) and dizziness (2 [4.08%]) (Table 4). Dyskinesia in both of the patients resolved after PPX dose reduction in one and taking PPX and LB separately in the other. Another patient experienced occasional dizziness and nausea, and his symptoms ameliorated after switching to one tablet of extended-release PPX at night.

All of the TEAEs were mild and tolerable, and none of the patients discontinued the treatment due to TEAEs (Table 4).

**Table 4:** Treatment-emergent adverse events (TEAEs) (SS).

TEAEs	LCE (*N* = 79)	PPX (*N* = 49)
Any TEAEs, *n* (%)	6 (7.59%)	7 (14.29%)
Types of TEAEs, *n* (%)		
Urine discoloration	3 (3.80%)	0
Dyskinesia	1 (1.27%)	2 (4.08%)
Insomnia	1 (1.27%)	0
Dizziness	1 (1.27%)	0
Constipation	0	1 (2.04%)^a^
Chest discomfort	0	1 (2.04%)^a^
Somnolence	0	1 (2.04%)
Restlessness	0	1 (2.04%)
Dizziness	0	2 (4.08%)^b^^,^^c^
Nausea	0	1 (2.04%)^b^
Blurred vision	0	1 (2.04%)^c^

a One patient reported both constipation and chest discomfort.

b One patient reported both dizziness and nausea.

c One patient reported both dizziness and blurred vision.

SS, safety set, LCE, levodopa/carbidopa/entacapone, PPX, pramipexole.

## 4 Discussion

In this multicenter, prospective, randomized, open-label, observational study, we compared the efficacy, safety and tolerability of LCE with LB plus PPX in LB-treated Chinese patients with PD who experienced WO. To the best of our knowledge, this is the first study assessing LCE treatment in Chinese patient with WO and is also the first study that compared LCE with PPX add-on in treating patients with WO.

Our study found that LCE and LB + PPX led to similarly significant shortening of daily OFF time and significant increase in the daily ON time in Chinese patients previously treated with LB who experienced WO. Specifically, LCE shortened the patient’s daily OFF time by 1.76 h and added 1.62 h to their daily ON time after 8 weeks of treatment. Our finding was consistent with previous studies (11–13, 26). In the Nomecomt study, adding 200 mg entacapone to each daily dose of levodopa taken by patients with WO increased their daily ON time by 1.2 h and decreased their daily OFF time by 1.3 h after 6 months of treatment and the mean daily levodopa dose was reduced by 12% (13). In the Celomen study, patients who experienced WO on levodopa treatment had a 1.5 h shorter daily OFF time and a 1.7 h longer daily ON time after taking 200 mg entacapone with their daily doses of levodopa for 6 months and their daily levodopa dose was reduced by 54 mg (12). In another RCT on LCE conducted in Korea, patients who switched directly to LCE had shortened daily OFF time (0.97 h and 1.25 h) and increased daily “ON” time (1.03 h and 0.90 h) at both a maintained and a reduced levodopa dose, respectively (26). In addition, consistent with our findings of improved WO symptoms by LCE, in the SENSE study, direct switch from levodopa/DDCI to LCE in patients with WO led to improved WO symptoms according to the WOQ-9, wherein tremor, any stiffness and mood change had the highest rate of improvement (15).

LEDD for patient switching to LCE in our study had a significant increase of 174.18 mg compared with baseline. As it has been reported that every dose of levodopa was 33% more effective when taken with entacapone (27), and as we did not reduce levodopa dose in our patients when they switched to LCE, a significant increase in LEDD was to be expected. Whether to reduce levodopa dose when switching to LCE has been debated. As adding entacapone to levodopa increases its plasma concentration by 20–50%, its bioavailability by 35% and its potency by 33% (1, 4, 27), it seems reasonable to reduce daily levodopa dose when switching to LCE. Furthermore, it has been suggested that switch to LCE without levodopa dose reduction increased the incidence of dyskinesia (27, 28). On the other hand, it has been reported that switch to LCE without levodopa dose reduction had significantly better effect on patient global impression of change (PGI) than switch with dose reduction, and it has also been suggested that reducing levodopa dose when switching to LCE had no advantage (9, 26). In our study, only one patient experienced mild dyskinesia that subsequently resolved after decreasing LCE dosing frequency. Therefore, direct switch to LCE without dose reduction is feasible and tolerable in our study.

Our study found that LB + PPX treatment shortened the patients’ daily OFF time by 1.51 h, prolonged their daily ON time by 1.38 h and improved their WO symptoms after 8 weeks of treatment. PPX is a nonergot DA widely used to prevent, delay and treat WO motor complications (3, 17, 28–30). As it directly stimulates the D_2_/D_3_ dopamine receptors in the brain and thus bypasses the degenerating neurons in the SN, its efficacy does not depend on conversion of levodopa to dopamine (17). With its long half-life (8 h in healthy people and 12 h in people >65 years old), it stimulates the D2/D3 receptor stably in a near physiological pattern (17). A RCT assessing PPX adjunct therapy in levodopa-treated patients with WO revealed that PPX adjunct treatment at a daily dose of 4.5 mg decreased the OFF time by 12%, increased the ON time by 2.5 h, improved UPDRS sum score of Part 2 (motor aspects of experiences of daily living) and Part 3 (motor examination) by 30% and reduced levodopa daily dose (31). The study further reported that the efficacy and safety of PPX were maintained over several years (31). Another 32-week RCT found that in levodopa-treated patients with motor fluctuations, PPX adjunct therapy at a dose of 4.5 mg/day decreased the daily OFF time by 31% and the levodopa daily dose by 27%, and that it improved motor functions and decreased PD severity during the “ON” and “OFF” times (32). Pinter et al., an 11-week RCT assessing PPX add-on in patients with WO motor complications, also reported that PPX add-on (5 mg/day) reduced the daily OFF time by 12% and added 1.7 h to the daily ON time at a maintained daily levodopa dose (33). Our findings were consistent with these previous studies.

There was 93.86 mg increase in the LEDD for patients receiving LB + PPX compared with baseline. As 1 mg of PPX is approximately equivalent to 100 mg of levodopa (27), the daily PPX dose in our study was around 1 mg, which was much lower than the 4.5 mg/day commonly adopted by western countries (17, 18, 31, 32). The PPX dose adopted in our study was consistent with the observations that Chinese patients often take PPX at a lower dose than those recommended by the Chinese PD consensus as well as than patients in the western countries (17, 18), Our study confirmed that PPX add-on was effective even at a low daily dose.

Our study further found that LCE and LB + PPX led to comparably significant improvement in the PDQ-39, PDSS-2 and BDI scores. Previous studies also found that LCE treatment improved PDQ-39, PDSS-2 or PDSS, and BDI scores significantly, as did PPX (14, 21–23, 26, 34–36). The PDQ-39 is the most widely use, PD-specific QoL questionnaire that is sensitive to various aspects of changes in a patient’s life (10, 21, 35), the PDSS-2 is a reliable tool to evaluate sleep-related response to treatment in patients with PD (22), and the BDI is a recommended scale for assessing severity of depression in patients with PD (37). Therefore, our findings, along with those of previous studies, suggested that both LCE and PPX add-on could improve sleep quality, ameliorate depression and improve QoL in patients with PD who experienced WO. The fact that dopamine is an important factor in circadian regulation may play a role in sleep improvement by LCE and PPX (22, 36). Depression is present in 40–70% of PD cases, and both dopaminergic pathway dysfunction and PD-related motor complications may cause depression (17, 37). Improvements in WO symptoms, sleep quality as well as depressive symptoms all could help to improve QoL in a patient. One caveat here was that the LCE group had a significantly lower baseline PDSS-2 score than the PPX group in our study. Therefore, our observation that the two groups of patients had comparable improvements in their PDSS-2 scores should be interpreted with caution. On the other hand, the difference in the baseline PDSS-2 scores between the two groups would not affect our observation that both treatments significantly improved the patients’ PDSS-2 scores.

Over 90% of the patients in both groups reported improvement after 8 weeks of treatment according to the CGI-C, demonstrating good efficacy of both treatments. Our results were in line with previous findings (15, 16, 37). In the SENSE study, the CGI-C indicated that 82.1% of the patients who directly switched from LB to LCE reported improvement in their symptoms (15), while in the TC-INIT trial, according to the CGI-C, 73% of the patients who switched from levodopa/DDCI to LCE indicated that their symptoms improved after 6 weeks of treatment (16). As to PPX, Mizuno et al. reported that 61.8% of the patients who received PPX add-on to their levodopa/DDCI regimen reported improvement after 12 weeks of treatment according to the CGI (37).

Both treatments were safe and tolerable, with 7.59% of the patients in the LCE group and 14.29% of the patients in the PPX group reporting mild TEAEs. Urine discoloration, dyskinesia, insomnia and dizziness reported by patients in the LCE group has all been previously reported (15, 21, 26). In addition, the TEAEs reported by patients in the PPX group are all common TEAEs of PPX (17, 31–33). The incidences of TEAEs of both treatments were lower than previously reported (15, 24, 31–33). As there was only one mandated follow-up visit during the 8-week treatment in our study, it is possible that some TEAEs were not collected. Although no patient reported hallucination as a TEAE, an examination of the UPDRS, Part 1 revealed significant difference in changes in the hallucination and psychosis sub-score between the two treatments, wherein LCE slightly decreased the hallucination and psychosis sub-score, while PPX increased it slightly. This is not surprising, as hallucinations are common TEAEs in patients receiving long-term DA treatment (17, 31, 32). DA treatment was widely used for Chinese patients with WO, and it has been reported that DA use was an independent risk factor for visual hallucination in Chinese patients with PD (38). Therefore, it is important to monitor the occurrence of hallucination in patients receiving long-term PPX treatment. Clozapine was effective in reducing hallucination in patients with PD (38, 39), and it is a commonly used treatment for hallucination in Chinese patients with PD.

One major limitation of the study is that the number of patients enrolled in the study was substantially less than the planned sample size for the study. As a result, the statistical power of the study was reduced. Reasons for the modest sample size included strict inclusion/exclusion criteria, public’s limited understanding of our research, concerns over its methods and integrity, burden on the patients and their caregivers and the COVID pandemic. As a result, the recruitment process was extremely slow despite our best efforts to circulate the information about the study in hospitals and patients communities and to communicate with and educate eligible patients and their caregivers about the study and alleviate their concerns. Finally, the recruitment was halted during the height at COVID pandemic.

The study has several other limitations. First, it is a short-term study, and as such, long term efficacy, safety and tolerability of LCE and LB + PPX could not be determined from the study. Previous long-term studies (up to 5 years) of LCE or levodopa/DDCI in combination with entacapone reported that the treatment was generally safe and well tolerated, and that most of the dopaminergic TEAEs (aggravation of Parkinsonism, dyskinesia and nausea) occurred during the first 4 weeks of the treatment and could often be managed by levodopa dose reduction (1, 40, 41). However, it has also been reported that LCE was associated with increased risk of dyskinesia compared with LC after 134 weeks of treatment (42). Other non-dopaminergic TEAEs such as diarrhea were spread pretty evenly over the treatment period (40–42). As to PPX, a 4-year study of PPX treatment in patients with advanced PD reported that its profile of TEAEs was consistent with the safety profiles of DAs and that the most common TEAEs were dyskinesia, dizziness, insomnia, hallucination and asymptomatic orthostatic hypotension (43). It also reported that the prevalence of aggravation of Parkinsonism, hallucination, pain and confusion increased over time (43). Long-term use of PPX was also associated with increased risk of developing impulse control disorder (44, 45). Second, as this is an open-label study, it is possible that there was patient bias and/or investigator bias. A patient’s perception of the effectiveness of LCE or PPX could be affected by his/her knowledge of the treatment, and interpretation of the results could be affected by an investigator’s knowledge and expectation for the treatment. Third, as there was only one mandated follow-up visit at the end of the study, it is possible that some TEAEs were missed. Fourth, as all of the patients in the study experienced WO on stable LB treatment that lowered their QoL, our study did not include a group of patients on LB only treatment as control out of ethical consideration. This might reduce the strength of causal inference in our study. Instead, our study was designed to compare the two treatments as well as improve the patients’ conditions, so that treatment optimization could be explored. The fact that changes in the main outcome measures, the “ON” and the “OFF” time from baseline in the two treatment groups were consistent with previous study confirmed the effectiveness of both treatment regimens. The strengths of the study are as follows. First, as a multicenter study, it allows for improved reproduction and generalization. Second, besides changes in the ON and the OFF time and WO symptoms, the study also assessed changes in QoL, sleep quality and depression in the patients to ensure a more comprehensive efficacy analysis.

In conclusion, LCE and LB + PPX were both effective, safe and tolerable in treating Chinese patients with PD who experienced WO. Both treatments could improve WO motor fluctuations, decrease the OFF time and increase the ON time, and improve QoL in the patients.

## Data Availability

The raw data supporting the conclusions of this article will be made available by the authors, without undue reservation.
