# Supplementary material for: Levodopa/carbidopa/entacapone versus levodopa/benserazide plus pramipexole in Chinese patients with Parkinson’s disease experiencing wearing off
Source: Front Neurol. 2025 Nov 19;16:1682614. doi: 10.3389/fneur.2025.1682614 (PMC12672222; doi:10.3389/fneur.2025.1682614)
Supplement: Supplementary file 1 [file Table_1.DOCX]

***Supplementary material***

**Supplementary Text 1 Participating Hospitals**

1) The Brain Hospital Affiliated to Nanjing Medical University;

2) BENQ Medical Center;

3) Affiliated Zhongda Hospital of Southeast University;

4) Nanjing Hospital of Chinese Medicine Affiliated to Nanjing University of Chinese Medicine;

5) The Second Affiliated Hospital of Soochow University;

6) Changshu Hospital affiliated to Nanjing University of Chinese Medicine;

7) Affiliated Hospital of Nantong University;

8) The Second Affiliated Hospital of Xuzhou Medical University;

9) The Affiliated Suqian Hospital of Xuzhou Medical University;

10) Jiangsu Province (Suqian) Hospital;

11) Huaibei People’s Hospital; and

12) The First Affiliated Hospital of Anhui Medical University.

**Supplementary Table 1** Schedule of switching to LCE for patients whose daily levodopa dose was >300mg (but ≤600mg) during the 4-week titration period

| Titration | Morning dose | Noon dose | Evening dose |
| --- | --- | --- | --- |
| Week 1 | LCE+LB | LB | LB |
| Week 2 | LCE+LB | LCE+LB | LB |
| Week 3 | LCE+LB | LCE+LB | LCE+LB |
| Week 4 | LCE+LB | LCE+LB | LCE+LB |

Abbreviations: LCE, Levodopa/Carbidopa/Entacapone (one tablet); LB, Levodopa/ Benserazide
